# Ivabradine and Blood Pressure Reduction: Underlying Pleiotropic Mechanisms and Clinical Implications

**DOI:** 10.3389/fcvm.2021.607998

**Published:** 2021-02-10

**Authors:** Fedor Simko, Tomas Baka

**Affiliations:** ^1^Institute of Pathophysiology, Faculty of Medicine, Comenius University, Bratislava, Slovakia; ^2^3^*rd*^ Department of Internal Medicine, Faculty of Medicine, Comenius University, Bratislava, Slovakia; ^3^Institute of Experimental Endocrinology, Biomedical Research Center, Slovak Academy of Sciences, Bratislava, Slovakia

**Keywords:** ivabradine, blood pressure, endothelial dysfunction, vascular stiffness, neurohumoral activation

## Introduction

Elevated heart rate (HR) is a well-recognized but somewhat neglected risk factor among the healthy population and various cardiovascular pathologies (1). High HR is fraught with a spate of detrimental cardiovascular consequences including immense myocardial oxygen demand in reduced diastolic perfusion time (2) and low, oscillatory vascular shear stress with high tensile stress triggering endothelial dysfunction (3). Although beta-blockers (BBs) are considered to be the cornerstone treatment of elevated HR in various cardiovascular pathologies, they are associated with negative inotropy, a number of side effects, and undesirable metabolic actions limiting their usage (4, 5). Thus, new approaches to HR reduction are being continuously sought out.

The inhibition of the I_f_ current in the sinoatrial node (SAN) seems to offer a promising approach to the reduction of elevated HR. Indeed, the SAN's pacemaker cells are inherently capable of cyclic variations of the resting membrane potential necessary for spontaneous depolarization. The SAN's spontaneous slow diastolic depolarization is administered by a mixed sodium/potassium inward current, known as an I_f_ current, through the “funny” (f)-channel (6). Structurally, the f-channel belongs to hyperpolarization-activated, cyclic nucleotide-gated (HCN) channels and is activated by both hyperpolarization in the diastolic voltage range and intracellular cyclic adenosine monophosphate (7). Ivabradine selectively inhibits the I_f_ current, thus reducing the steepness of SAN's diastolic depolarization, ensuing diastole prolongation without affecting action potential duration or inducing negative inotropy (7, 8).

Several studies have assessed ivabradine's efficacy in clinical settings. In the SHIFT study, the investigation of 6,558 patients with systolic heart failure (HF) during a median 22.9 month follow-up period revealed that the addition of ivabradine to an established HF therapy significantly reduced the primary composite endpoint of hospital admission for worsening HF or cardiovascular death. Considering the results of the SHIFT study, ivabradine is recommended for patients with systolic HF and HR above 70 bpm despite an evidence-based optimal medical therapy (with or without BB) to reduce the composite endpoint of hospitalization and mortality (9, 10). The BEAUTIFUL study comprised 10,917 systolic HF patients with HR above 70 bpm suffering from stable coronary artery disease (CAD), and the primary endpoint was a composite of cardiovascular death and hospital admission for acute myocardial infarction or HF. Although neither the primary endpoint nor the cardiovascular death rate improved, ivabradine reduced the secondary endpoints of hospital admissions for myocardial infarction and coronary revascularization (11). However, in the SIGNIFY study involving 19,102 patients with stable CAD but without HF, ivabradine did not reduce the compound primary endpoint of cardiovascular death and myocardial infarction (12).

Hypertension, however, is a substantially different condition, and data regarding ivabradine's effect on peripheral blood pressure (BP) in a hypertensive population are scanty. Yet ivabradine's interference with central BP (CBP) was indicated in several studies. Lopatin and Vitale (13) reviewed five studies analyzing ivabradine's effect on CBP in patients with CAD: two studies reported a neutral effect, while in two other studies and in one study, ivabradine decreased and increased CBP, respectively. In 12 normotensive patients with stable CAD and HR ≥ 70 bmp, a 3 week ivabradine treatment reduced brachial systolic and diastolic BP, while the HR reduction did not increase central aortic BP (14). Moreover, in patients with arterial hypertension and CAD treated with ivabradine, the increase in HR between resting conditions and early recovery post exercise showed a trend toward correlation with the radial augmentation index (15).

Besides ivabradine's HR-reducing action, which is considered to be a principal mechanism of its benefit, ivabradine exerts a number of pleiotropic effects, some of which may partly be HR independent (16, 17) and some of which are still emerging.

## Ivabradine and Bp Reduction

BBs have been a well-established means for HR reduction and the improvement of the energetic state of the myocardium in various cardiovascular diseases (18). The important advantage of ivabradine over BB seems to be its apparent independence from the sympathetic nervous system, thus avoiding negative inotropy or alpha-adrenoceptor-mediated coronary vasoconstriction (17).

According to generally accepted assumptions, ivabradine exerts a neutral effect on arterial BP in both experimental and clinical settings (8–12). However, based on several recent pieces of evidence, ivabradine could reduce BP under certain conditions:

- In an experiment with N^G^-nitro-l-arginine methyl ester (l-NAME)-induced nitric oxide-deficient hypertension in rats, ivabradine (10 mg/kg/day) reduced HR and systolic BP measured by non-invasive tail-cuff plethysmography during a period of 4 weeks. Systolic BP was reduced from the first week by ivabradine treatment and continued to decrease each week. In the fourth week of the experiment, ivabradine reduced systolic BP by 15%, and the 4 week average systolic BP was decreased by 8% via ivabradine compared to that in the l-NAME group (19). In another study with l-NAME-induced hypertension, ivabradine reduced systolic BP not only in the l-NAME group (by 21%) but even in the control group (by 26%) (20).
- In a study that sought to improve non-dipping HR in a rat model of l-NAME-induced hypertension, daytime and nighttime systolic BP and HR were measured weekly after administration of the daily dose of ivabradine (10 mg/kg/day) at either daytime or nighttime during a period of 4 weeks. Interestingly, both daytime- and nighttime-dosed ivabradine decreased both daytime and nighttime systolic BP in hypertensive rats each week, reaching the largest 14% systolic BP decline during the last week of the experiment (21).
- In the three rat models of acute stress induced by handling (mild stress), restraint (moderate stress), or immobilization (severe stress), ivabradine (5 mg/kg) was administered intraperitoneally 30 min before stress exposure. In the groups pretreated with ivabradine, lower values of HR and mean arterial BP were observed in the baseline period, during exposure to stressors, as well as during the rest period following stress exposure in all three types of stressors applied and all intervals investigated (22).
- Two studies assessed the effect of acute or chronic ivabradine on HR and BP in spontaneously hypertensive rats and Wistar–Kyoto controls as measured by carotid catheterization under pentobarbital anesthesia. The acute administration of four consecutive ivabradine doses (1 mg/kg, i.v.) decreased systolic, diastolic, and mean BP in hypertensive rats and in controls (except for systolic BP, which remained unchanged) and increased pulse pressure in both rat strains (23). The chronic, 28 day administration of ivabradine (8.4 mg/kg/day via subcutaneous osmotic minipump) decreased systolic, diastolic, and mean BP and increased pulse pressure in both rat strains (24).
- In healthy volunteers treated with ivabradine (30 mg), propranolol (40 mg), or a placebo, hemodynamic parameters were investigated at rest and before and during tilt and exercise tests 2 and 5 h after drug intake. Ivabradine significantly reduced systolic BP at rest. However, during tilt and exercise tests, only propranolol but not ivabradine reduced systolic BP (25).

The mechanisms underlying the ambiguity of ivabradine's effect on BP in different conditions remain elusive. Yet the following two factors might be considered determining: (i) the pathophysiology of the ivabradine-treated disease, as in a rat model of isoproterenol-induced HF, ivabradine prevented detrimental systolic BP decline indicative of improved cardiac function (26), while in a rat model of l-NAME-induced hypertension, ivabradine decreased systolic BP by exerting antihypertensive properties (19); and (ii) concomitant therapy, as in pivotal clinical studies, e.g., SHIFT, BEAUTIFUL, or SIGNIFY, ivabradine was administered on top of the evidence-based optimal medical therapy, often including drugs modulating the sympathetic nervous system and/or renin–angiotensin–aldosterone system (8–12), thus presumably giving minimal space for ivabradine to exert an effect on BP. In clinical studies with HF patients, HR reduction without affecting BP was considered to be desirable, since the BP-reducing effect of well-established HF therapeutics such as BB, angiotensin-converting enzyme inhibitors (ACEis), angiotensin II type 1 receptor blockers (ARBs), or mineralocorticoid receptor antagonists (MRAs) can limit the achievement of the target doses. Interestingly, it has been shown that the efficacy and safety of ivabradine in HF patients were independent of systolic BP (27).

## Potential Mechanisms Behind Ivabradine's Interference With Bp

**Figure 1:**
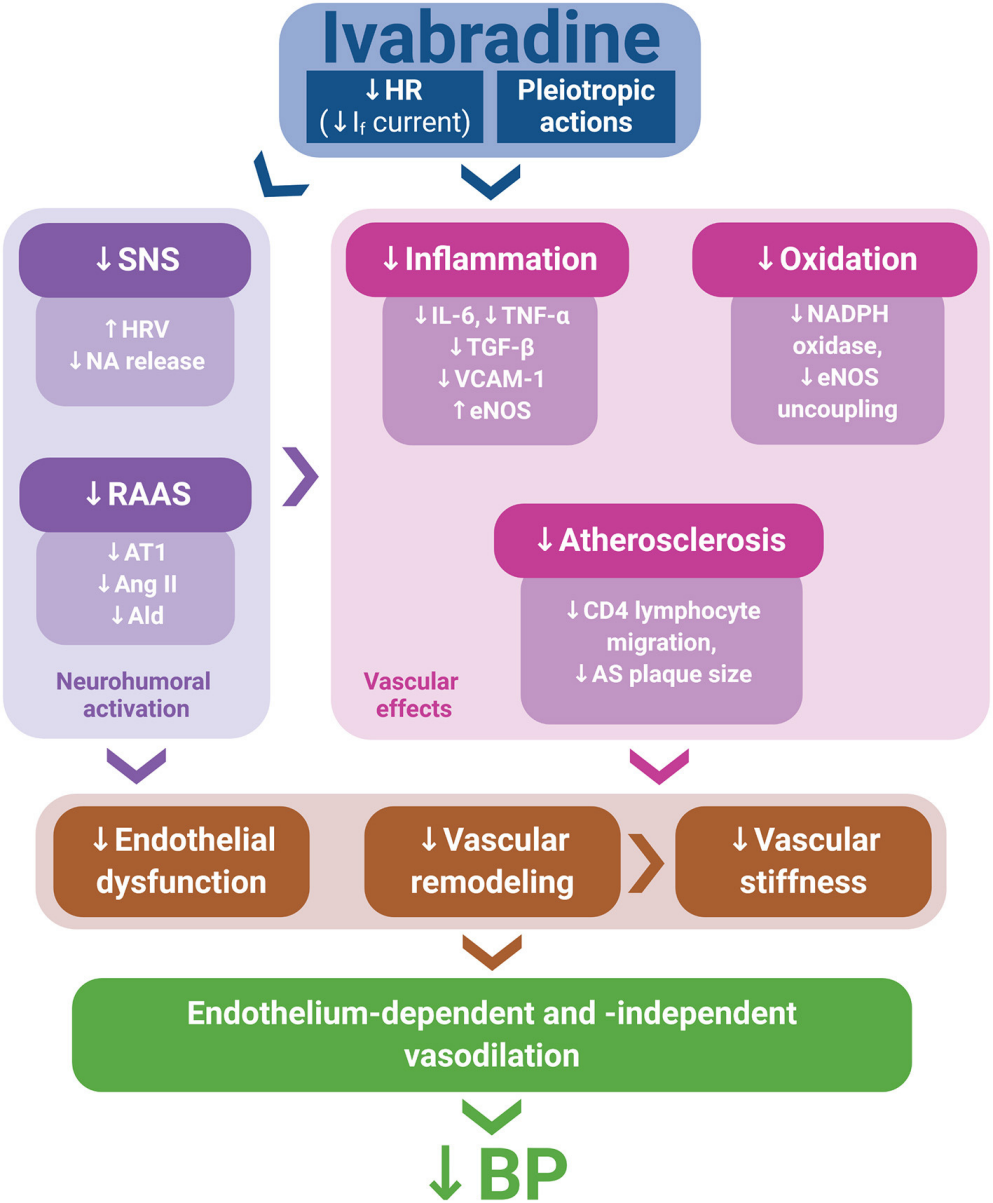
Ivabradine's pleiotropic actions, along with heart rate (HR) reduction via I_f_ current inhibition, might mitigate neurohumoral activation, inflammation, oxidative stress, and atherosclerosis development, thus improving endothelial function and attenuating vascular remodeling and stiffness. These effects may contribute to endothelium-dependent and endothelium-independent vasodilation, potentially resulting in blood pressure (BP) reduction. Ald, aldosterone; Ang II, angiotensin II; AS, atherosclerotic; AT1, angiotensin II type 1 receptor; eNOS, endothelial nitric oxide synthase; HRV, heart rate variability; NA, noradrenaline; RAAS, renin–angiotensin–aldosterone system; SNS, sympathetic nervous system.

Although experimental data on ivabradine's BP-reducing effect are scarce and large prospective clinical studies featuring ivabradine and a hypertensive population are lacking, numerous potential mechanisms contributing to the BP reduction by ivabradine in experiments demonstrated in this study could be considered. Indeed, several pleiotropic effects of ivabradine, such as anti-inflammatory and antioxidant actions, the improvement of endothelium-dependent and endothelium-independent vascular relaxation, anti-atherosclerotic effects, and the attenuation of the neurohumoral activation, might individually or in concert contribute to BP reduction (Figure 1).

### Ivabradine's Interference With Oxidative Stress, Inflammation, Endothelial Dysfunction, and Vascular Stiffness

Several plausible explanations for the potential BP-reducing effect of ivabradine are emerging. Besides HR reduction, ivabradine was shown to exert manifold pleiotropic effects within the vasculature in terms of inflammation and oxidative stress reduction and improvement of endothelial function and vascular elasticity (28). Indeed, in low-shear-stress-damaged isolated endothelial cells, ivabradine prevented inflammation and oxidative stress via the TOR/eNOS pathway (29). Ivabradine reduced reactive oxygen species levels in atherogenic diet-induced hypercholesterolemic rabbits (30). In apolipoprotein E-knockout mice, ivabradine reduced NADPH oxidase activity and prevented eNOS uncoupling (31); decreased monocyte chemotactic protein mRNA, markers of superoxide production and lipid peroxidation, and atherosclerotic plaque size (32); reduced the aortic mRNA expression of IL-6, TNF-α, and TGF-β (33); and downregulated pro-apoptotic and pro-inflammatory genes (23, 34). In hypercholesterolemic mice, ivabradine reduced the expression of pro-inflammatory VCAM-1 and enhanced the expression of anti-inflammatory eNOS on the inner surface of the aorta (35). These potentially protective effects of ivabradine could result in the improvement of aortic elasticity and endothelium-dependent relaxation (30, 33, 36). Indeed, ivabradine reduced neointimal hyperplasia and intima–media ratio in hypercholesterolemic rabbits (30) and attenuated aortic fibrosis and stiffness in diabetic mice (37). Ivabradine also inhibited the chemokine-induced migration of CD4-positive lymphocytes, thus potentially curbing atherosclerosis development (38). In clinical settings, ivabradine improved aortic elasticity and endothelial function in chronic systolic HF (36) and fostered the flow- and nitroglycerin-mediated dilation of the brachial artery in patients with CAD (39). Similarly, in patients with chronic stable CAD, HR reduction by ivabradine improved flow-mediated vasodilation and reduced the arterial stiffness of the brachial artery (40).

Increasing the magnitude of wall shear stress via HR reduction seems to be the underlying mechanism of ivabradine's arterial protection (35). Moreover, ivabradine increased the brain capillary density in mice with chronic mental stress (41), enhanced capillary formation in mice with myocardial infarction (42), and improved coronary reserve in rats afflicted with myocardial infarction potentially by the reduction of periarteriolar collagen (43). Taken together, these findings suggest that ivabradine may improve endothelium-dependent and endothelium-independent vascular relaxation, resulting in vasodilation along with improvement of microcirculation, thus contributing to BP reduction and improved organ perfusion.

Ivabradine was also shown to exert cardioprotection by the attenuation of both apoptosis and matrix metalloproteinase expression (44), to improve mitochondrial respiration, and to enhance ATP production and calcium retention capacity independent of HR reduction (16). Moreover, ivabradine showed a positive inotropic action induced by enhanced sarcoplasmic/endoplasmic reticulum calcium ATPase 2a (SERCA2a) activity (45). Thus, the vascular and cardiac protective pathways, along with HR reduction, may underlie ivabradine's effects of cardiovascular benefit.

### Ivabradine Modifies Neurohumoral Pathways

The potential relation of ivabradine to neurohumoral systems should be taken into account. Although ivabradine is considered to exert its principal protection as a direct and selective HR reducer via inhibition of the I_f_ current in the SAN, its potential interaction with the sympathetic nervous system cannot be excluded. In the above-mentioned experiment with three acute stress rat models, the reduced BP in ivabradine-pretreated rats exposed to handling stress was associated with reduced adrenaline and noradrenaline release into the blood stream compared to placebo treatment (22). Furthermore, in Dahl salt-sensitive rats, chronic ivabradine treatment reduced mortality along with the reduction of urinary noradrenaline excretion (46). In a rat model of doxorubicin-induced HF, the measuring of HR variability indicated an ivabradine-induced improvement of the autonomic imbalance (47). In substudies of large clinical trials, the HR variability analysis has shown an ivabradine-mediated shift toward a parasympathetic tone (48, 49); and in hypertensive patients with metabolic syndrome, ivabradine reduced sympathetic activation (50).

Similarly, data regarding ivabradine's interaction with the renin–angiotensin–aldosterone system are emerging. In ApoE-deficient mice, ivabradine reduced the serum level of angiotensin II (Ang II) (51), reduced the mRNA expression and protein of the Ang II type 1 receptor (AT1 receptor) (33), and downregulated Ang II-regulated pro-inflammatory genes (34). Moreover, in rats with myocardial infarction, ivabradine reduced the myocardial protein expression of the AT1 receptor (43, 52) and tissue angiotensin-converting enzyme (52). In l-NAME-induced hypertension, along with BP reduction, ivabradine reduced the serum concentration of aldosterone and the aldosterone/Ang II ratio (19). Blunting the sympathetic nervous system or the renin–angiotensin–aldosterone system may contribute to the potential BP-reducing effect of ivabradine via the reduction of peripheral artery resistance or circulating volume.

## Concluding Remarks and Perspectives

In the SHIFT study, ivabradine created hope for the treatment of HF patients with elevated HR. However, some studies with ivabradine were neutral and did not meet expectations. Thus, the indication for ivabradine should be considered carefully (53). On the other hand, the unique nature of ivabradine could be considered in several off-label indications (54), such as inappropriate sinus tachycardia (55) or postural orthostatic tachycardia syndrome (56). Ivabradine was recently shown to improve hypertensive heart function in rats with l-NAME-induced hypertension (19).

Hypertension with elevated HR might be another indication for ivabradine (57). Increased HR in hypertension is an undesirable condition that worsens the prognosis; thus, the decision regarding the optimal treatment of elevated HR in hypertension is an issue at the crossroads and has attracted professional attention for decades (1, 58). Ivabradine could become a candidate for this indication, considering a number of its pleiotropic effects:

- Based on several examples presented in this work, ivabradine might be able to reduce BP and could contribute to the reduction of the hemodynamic burden in hypertension. According to a scientific statement from the American Heart Association on the detection, evaluation, and management of resistant hypertension, the number of patients with resistant hypertension is expected to significantly increase (59, 60); therefore, seeking new approaches to BP control will be of utmost importance (61, 62).
- It has been previously shown that besides the increase in daily HR mean, insufficient HR decline during bedtime, i.e., non-dipping HR, increases cardiovascular risk (63–65). Moreover, non-dipping HR seems to be more frequent in hypertensive patients with chronic kidney disease than in the hypertensive population without kidney affliction (66). HR reduction with ivabradine reaches its peak effect in 3 to 4 h and lasts 8 to 12 h after ingestion (67), thus subjecting ivabradine to a flexible dosing scheme for targeting mean HR or nighttime HR. A well-tailored dosing of ivabradine might reverse non-dipping HR to a desirable HR dipping pattern (21, 57), thus presumably further reducing cardiovascular risk in hypertension.
- As opposed to BB (4, 5), ivabradine has not been observed to have negative metabolic effects (68, 69), which would be beneficial for hypertensive patients with metabolic syndrome prone to dyslipidemia, hyperuricemia, or diabetes mellitus.
- Ivabradine does not induce anxiety or other behavioral disorders in rats (70, 71), whereas BB therapy was shown to be associated with psychological disorders, such as nightmares (72, 73).
- Ivabradine was found to exert hypertensive heart protection. Indeed, in l-NAME-induced hypertension, ivabradine improved the systolic and diastolic dysfunctions of the remodeled left ventricle (LV) (19); and in a transverse aortic constriction mouse model, ivabradine reduced LV hypertrophy, fibrosis, and cardiomyocyte apoptosis and improved LV function (74). In a pig model of chronic Ang II infusion-induced hypertension and diastolic LV dysfunction, the acute administration of ivabradine improved LV filling parameters by an HR-independent mechanism (75). Moreover, ivabradine exerted an antihypertrophic effect on the aorta in spontaneously hypertensive rats (24) and renoprotection in rats with l-NAME-induced hypertension (76).

Taking into account ivabradine's HR- and (potential) BP-reducing effects associated with target organ protection and the lack of undesirable metabolic and behavioral consequences (often seen with BB), it appears reasonable to suggest the consideration of ivabradine for hypertensive patients with elevated HR, especially for those co-afflicted with metabolic disorders.

## Author Contributions

FS conceived and drafted the manuscript. TB revised the manuscript. Both authors participated in data analysis and interpretation and approved the submitted version.

## Conflict of Interest

The authors declare that the research was conducted in the absence of any commercial or financial relationships that could be construed as a potential conflict of interest.
